# A ‘Moore's law’ for fibers enables intelligent fabrics

**DOI:** 10.1093/nsr/nwac202

**Published:** 2022-09-24

**Authors:** Shengtai Qian, Mingyang Liu, Yuhai Dou, Yoel Fink, Wei Yan

**Affiliations:** School of Electrical and Electronic Engineering, Nanyang Technological University, Singapore 639798, Singapore; School of Electrical and Electronic Engineering, Nanyang Technological University, Singapore 639798, Singapore; Institute for Energy Materials Science, University of Shanghai for Science and Technology, Shanghai 200093, China; Research Laboratory of Electronics, Massachusetts Institute of Technology, Cambridge, MA 02139, USA; School of Electrical and Electronic Engineering, Nanyang Technological University, Singapore 639798, Singapore; School of Materials Science and Engineering, Nanyang Technological University, Singapore 639798, Singapore

**Keywords:** functional fibers, fiber electronics and optoelectronics, intelligent fabrics, wearable electronics, fabric computation, human-interfaced bioelectronics

## Abstract

Fabrics are an indispensable part of our everyday life. They provide us with protection, offer privacy and form an intimate expression of ourselves through their esthetics. Imparting functionality at the fiber level represents an intriguing path toward innovative fabrics with a hitherto unparalleled functionality and value. The fiber technology based on thermal drawing of a preform, which is identical in its materials and geometry to the final fiber, has emerged as a powerful platform for the production of exquisite fibers with prerequisite composition, geometric complexity and control over feature size. A ‘Moore's law’ for fibers is emerging, delivering higher forms of function that are important for a broad spectrum of practical applications in healthcare, sports, robotics, space exploration, etc. In this review, we survey progress in thermally drawn fibers and devices, and discuss their relevance to ‘smart’ fabrics. A new generation of fabrics that can see, hear and speak, sense, communicate, harvest and store energy, as well as store and process data is anticipated. We conclude with a critical analysis of existing challenges and opportunities currently faced by thermally drawn fibers and fabrics that are expected to become sophisticated platforms delivering value-added services for our society.

## Teaser

A ‘Moore's law’ for fibers enables intelligent textiles that see, hear and speak, sense, communicate, harvest and store energy, store and process data, and eventually compute.

## INTRODUCTION

Fibers and fabrics hold great societal relevance and impact on our everyday lives [1–10]. They are an indispensable part of a broad spectrum of products from the clothes on our body to upholstery in rooms, and from composites to spacesuits of astronauts [11–14]. While ubiquitous, the use of fibers and fabrics as solely aesthetic and protective media has for the most part remained unchanged for millennia [15]. The modern electronics industry, on the other hand, has gained an expeditious development in which novel materials, fabrication methods and device architecture enable unprecedented optical, electronic and optoelectronic functions and unique applications of electronic products employed in our everyday lives. Motivated by the leap in the electronics industry as well as the importance of fabrics to humans—fabrics are thought of as the second skin on human bodies and are, thus, exposed to a vast amount of acoustic, optical, electrical, mechanical, physiological and biological signals emanating from the body that reflect individual health and disease status, and interaction with the environment—exploration of smart fabrics, a new generation of fabrics that augment wearers’ capability in personalized sensing, communication, healthcare, action and intellect (computation) started some years ago.

To functionalize fabrics, some early strategies were focused on the use of silicon-based electronics as they are high-performance, and their use and evaluation standards are well established. Such materials and devices are, however, difficult to be integrated with fabrics for reasons as follows. First, the well-established semiconductor processing techniques such as photolithography and nanoimprinting, typically used for fabricating 2D thin-film devices, are not applicable to flexible fabrics with highly curved geometries. Second, while configuring silicon into ‘wavy’ shapes and bonding to polymeric substrates leads to flexible and stretchable systems with performance of conventional wafer-based devices [16], this method still remains incompatible with traditional fabric-manufacturing technologies, such as weaving, knitting or knotting. Attaching rigid silicon-based devices or their planar films onto the fabric surface represents a route for smart fabrics. This technology, however, adds much of an additional burden on the wearer, which restricts the wearability, functions and practical use of the resulting fabrics.

A well-received strategy for the development of smart fabrics is to revolutionize materials and structure, and to impart functionality at the fiber or yarn level. So far, a breadth of techniques for the fabrication of smart fibers, such as wet spinning [17,18], dry spinning [19], blow spinning [20], electrospinning [21], melt spinning [4], physical and chemical vapor deposition [22], physical convolution and wrapping [23] and 3D printing [24] have been developed, resulting in smart fabrics capable of photo-sensing [25], deformation and motion sensing [26,27], chemical sensing [28], humidity sensing [29], healthcare monitoring [30], actuation [31,32], data communication [33], display [34], energy harvesting and storage [35,36], thermal regulation [37], data processing [33], etc.

Among the various methods for fiber fabrication, the preform-to-fiber thermal-drawing technique has emerged as an unprecedentedly compelling platform for enabling fibers to evolve into functional devices and smart systems [11]. The thermal-drawing technique was originally exploited to manufacture optical fibers for telecommunication. This approach starts with making a macroscopic preform, a scaled-up model of the fiber, that assembles various functional materials or prefabricated devices at a desired position. Subsequently the preform is fed into the furnace of the fiber-draw tower, where the preform is heated up to a high temperature at which the materials either soften or melt. Then an external force pulls the preform and, thus, all the materials end up flowing next to each other while keeping their relative positions and shrinking down in dimensions, yielding thin fibers with sophisticated architecture and complex functionalities. A ‘Moore's law’ for fibers, where fiber functions escalate in a predictable manner as more materials and devices are integrated into a single strand or multiple strands, is accordingly emerging [15]. Nevertheless, it is noteworthy that co-drawing different materials imposes constraints on the thermal, rheological and mechanical properties of fiber materials, which has been elaborated in a previous reference [11]. This approach is highly scalable. Drawing a tens-of-centimeter-long preform enables a thousands-of-meter-long fiber. Furthermore, drawing a preform consisting of multiple device architecture results in a fiber with a high density of devices and a variety of functionalities. Fibers that maintain the structure of the preform possess complex micro-structure and nanostructure. Thus, this is a powerful technique to bridge the length scale from nanometers to kilometers, and to deliver complex functionality across a multitude of length scales. The small cross section and large aspect ratio of the fiber allow its unique applications such as minimally invasive surgery and optical communications. More intriguingly, the scalability of the thermal-drawing approach is well matched to the scale of textile manufacturing processes in industry such as weaving, knitting and knotting, enabling large-scale manufacturing of smart fabrics using thermally drawn fibers [38].

**Figure 1.**
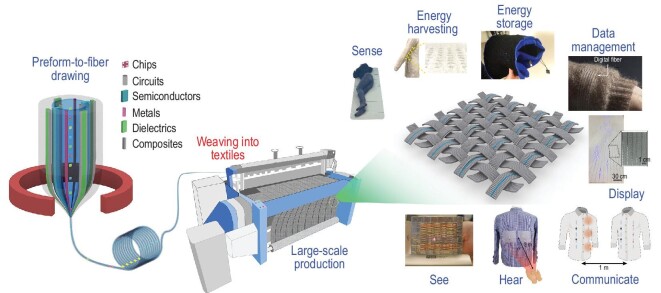
Thermally drawn fibers that integrate chips, circuits, semiconductors, metals, dielectrics, composites, etc. can be directly woven into fabrics enabling a multitude of functionalities. See: Reproduced with permission [25]. Copyright 2004, Nature Publishing Group. Hear: Reproduced with permission [38]. Copyright 2022, Nature Publishing Group. Communicate: Reproduced with permission [39]. Copyright 2018, Nature Publishing Group. Display: Reproduced with permission [39]. Copyright 2018, Nature Publishing Group. Sense: Reproduced with permission [40]. Copyright 2020, Wiley-VCH. Energy harvesting: Reproduced with permission [35]. Copyright 2020, Nature Publishing Group. Energy storage: Reproduced with permission [36]. Copyright 2020, Wiley-VCH. Data management: Reproduced with permission [33]. Copyright 2021, Nature Publishing Group.

In this review, we discuss ‘Moore's law’ for thermally drawn fibers enabling smart fabrics. We examine recent scientific and technological breakthroughs in materials, physics, design and fabrication strategies, and functions and applications of these smart fabrics. We highlight the new-generation fabrics that can see, hear and speak, sense, communicate, harvest and store energy as well as store and process data (Fig. 1) [25,33,35,36,38–40]. Finally, we present a critical analysis of these smart fabrics and our vision of future scientific and technological developments towards realizing computing fabrics that are expected to become sophisticated platforms delivering value-added services for our society.

## MECHANISTIC PLATFORMS EMPLOYED IN THERMALLY DRAWN FIBERS

To allow all integrated materials, such as conductors, semiconductors and insulators, to thermally flow next to each other during thermal drawing, some requirements on the thermal, rheological, chemical and mechanical properties of fiber materials should be met [41]. Despite these criteria, a wide range of functional materials have been incorporated into the platform, as summarized in Table 1. Many mechanistic platforms have been employed to produce functional fibers and fabrics.

**Table 1.** Typical functional fibers fabricated by thermal drawing that have been woven into smart fabrics.

Fibers	Mechanism	Active materials	Fabric applications	References
Optoelectronic fibers	Photoelectric effect	Chalcogenide semiconductors	Photo sensing, imaging	[25,59]
Optoelectronic fibers	Photoelectric effect	Semiconductor nanowires	Fluorescence imaging	[60]
Acoustic fibers	Piezoelectric effect	P(VDF-TrFE), Piezo-ceramics	Acoustic sensing and emission, communication	[38,61]
Diode fibers	PN junction theory, photoelectric effect	Silicon, germanium or gallium arsenide	Photo sensing, communication	[39]
Display fibers	PN junction theory	Silicon, germanium or gallium arsenide	Display	[39]
Temperature-sensing fibers	Temperature-triggered effect	Chalcogenide semiconductor	Temperature sensing	[62]
Temperature-sensing fibers	Thermoelectric effect	Thermoelectric materials	Temperature sensing, cooling	[52,63]
Triboelectric fibers	Triboelectric effect	SEBS, liquid metals	Breath sensing	[35]
Acoustic fibers	Piezoelectric effect	P(VDF-TrFE), piezo-ceramic	Heart-sound diagnosis	[38]
Stretchable fibers	Piezoresistive effect	SEBS, liquid metals	Mechanical sensing	[64]
Stretchable fibers	Capacitive effect	SEBS, liquid metals	Motion sensing	[26]
Soft fibers	Triboelectric effect	SEBS, liquid metals	Mechanical energy harvesting	[35]
Thermoelectric fibers	Thermoelectric effect	SnSe	Thermal energy harvesting	[53]
Supercapacitor fibers	Electrochemical effect	PVDF, LiTFSI^a^, carbon black	Energy storage	[36]
Battery fibers	Electrochemical effect	PVDF, lithium–iron–phosphate, lithium titanate	Energy storage	[65]
Digital fibers	PN junction theory	Silicon	Data storage and processing, logic thinking	[39]

^a^LiTFSI is the abbreviation for lithium bis(trifluoromethane sulfonimide).

### Photoelectric mechanism

Photosensitive semiconductors convert light into electricity. When the energy of the incident light is higher than that of the energy bandgap of the material, the electrons in the valence band absorb energy and jump to the conduction band. These excited electrons can move freely. Under an external voltage applied to the material, a photocurrent can be measured. Chalcogenide semiconductors are frequently harnessed in the low-temperature polymer platform [42]. Semiconductors with high melting points such as silicon or germanium are exploited in the high-temperature silica glass platform [43].

### PN junction

A PN junction is an interface or boundary formed between a p-type semiconductor and an n-type semiconductor. At the interface, it forms a depletion region acting as a barrier that prevents further flow of free electrons in the n-type semiconductor and holes in the p-type semiconductor. A PN junction allows the flow of current when forward biased and does not allow current flow when reverse biased. PN junctions underpin many electronic devices such as transistors, diodes, solar cells and LEDs. Micro-sized diodes, transistors and LEDs have all been integrated into the thermally drawn fibers for fabric sensing, communication and computation [33,39].

### Piezoelectric mechanism

Piezoelectric materials can generate electric charges when an external force is exerted on the materials. These materials can also produce mechanical deformation when an external voltage is applied on them. Piezoelectric polymers such as polyvinylidene difluoride (PVDF)-based materials have frequently been harnessed in the platform because of their low processing temperatures and mechanical flexibility [44,45]. Piezoceramics in the forms of micro- and nanoparticles are sometimes embedded into the piezo–polymer matrix to form a composite with enhanced performance. A recent study shows that the ceramic particles incorporated into the piezo–polymer matrix is not responsible for the drastically enhanced piezoelectric coefficient [38,46].

### Temperature-triggered mechanism

Fiber thermistors have been harnessed to sense small temperature variations. The core material is a chalcogenide semiconductor whose resistance is temperature-dependent [47]. The measured resistance can be described with an exponential form associated with the thermal activation energy of the material.

### Triboelectric mechanism

As a burgeoning energy-harvesting technology, triboelectric nanogenerators (TENGs) have been exploited in a myriad of applications. TENGs convert mechanical stimuli into electricity via contact-electrification and electrostatic-induction effects [48,49]. Many different configurations have been developed in the fiber platform for sensing, energy harvesting and powering [50,51].

### Thermoelectric mechanism

Thermoelectric materials can convert a temperature difference to an electric voltage and vice versa. Typical thermoelectric materials exhibit high melting points such that they can be co-drawn with silica-based glass materials [52]. The resulting fibers have been used for temperature sensing as well as cooling applications [52]. Laser-based thermal annealing is harnessed to control the crystal nucleation and growth for the fabrication of high-performance single-crystal thermoelectric fibers [53].

### Piezoresistive mechanism

Piezoresistive materials such as semiconductors and conductors change their electrical resistances when a mechanical strain is applied. A widely investigated system consists of conductive fillers dispersed in an insulating matrix. The conduction mechanism of such a composite can sometimes be described by the percolation theory where the transition from insulator to conductor occurs when a certain number of conductive fillers are dispersed [54]. Liquid metals and carbon material-based nanocomposites have been widely harnessed in the thermal-drawing platform for mechanical and motion-sensing applications [55].

### Capacitive mechanism

The capacitive effect exploits capacitance changes upon mechanical pressure applied on materials. The implementation of this mechanism into the fiber platform requires the integration of two electrodes. To trigger the capacitance change upon external stress stimulation, both the configuration of the soft claddings encapsulating electrodes and the configuration of electrodes separated by an air gap have been developed to monitor pressure and physiological signals [56,57].

### Electrochemical mechanism

Fiber batteries and supercapacitors store chemical energy and convert it into electrical energy. The current is produced by the reduction–oxidation reactions happening at the interface between electrodes and the electrolyte. The recent advance demonstrates that porous electrodes and electrolytes can be fabricated via the principle of polymer solution thermally induced phase separation during thermal drawing [58]. These porous structures expose new pristine surfaces and enhance ionic transport.

## FABRIC FUNCTIONALITIES

### Fabrics see

**Figure 2.**
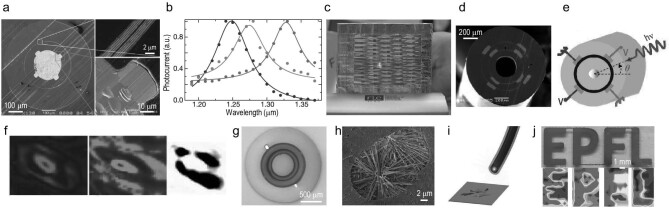
(a) Scanning electron microscopy (SEM) cross-sectional micrograph of the optoelectronic fiber with a 200-μm chalcogenide glass core surrounded by a poly ethersulfone (PES) cladding. The core region is surrounded by a resonant cavity structure. The semiconductor–metal interface is of excellent quality. (b) Measured photocurrent of the fiber device. (c) A woven optoelectronic fabric that detects the intensity and direction of the illumination light. (a–c) Reproduced with permission [25]. Copyright 2004, Nature Publishing Group. (d) An optoelectronic fiber integrating eight cascaded devices that enable the measurement of the intensity, direction and wavelength of incident radiation over a wide spectral range in the visible regime. (e) Schematic of the principle for the measurement of radiation direction. (f) Lensless imaging of a smiley face. (d–f) Reproduced with permission [69]. Copyright 2009, American Chemical Society. (g) Optical micrograph of the hybrid optoelectronic fiber integrating one optical fiber core and two photodetectors. (h) SEM cross-sectional micrograph of Se nanowires at the fiber tip. (i) Schematic of the fluorescence imaging system. (j) Fluorescent image of ‘EPFL’ obtained with the hybrid fiber. The logo is filled with dye Rhodamine B dissolved in ethanol. (g–j) Reproduced with permission [60]. Copyright 2017, Wiley-VCH.

The vision capability of humans relies on the detection and perception of light. Likewise, endowing fabrics with the ability to sense photons would enable them to see objects, which requires the integration of photosensitive semiconductors into the fibers of fabrics. A generation of optoelectronic fibers and fabrics has thus emerged [66,67]. The first thermally drawn optoelectronic fiber invented in 2004 contains an amorphous chalcogenide glass core (As_40_Se_50_Te_10_Sn_5_) intimately interfaced with four metallic electrodes (Sn), all of which are encapsulated within a transparent polyethersulphone polymer cladding [25]. The fiber intrinsically senses photons in a wide spectrum. To enable photodetection at a specific optical frequency, the optoelectronic domain is encircled by a photonic bandgap structure that only allows light with a specific wavelength to pass through and then to illuminate the photodetecting core (Fig. 2a). Such a unique design offers the possibility of narrowband photodetection (Fig. 2b). The resulting fiber is mechanically flexible and of small feature size, which allows one to weave it into a large-area optoelectronic fabric. The fabric web is capable of detecting the intensity of the external illumination via measuring the current flow through the metal–semiconductor–metal structure (Fig. 2c). Although a single fiber cannot determine the illumination position, a fabric web that consists of N horizontal fibers and N vertical fibers enables the localization of the photon stimulation via simply measuring the photocurrents of no more than 2 N fibers. This contrasts with the typical method where a N × N detector array should be established and it requires the measurement of N^2^ point elements. The principle based on fabric grids has also been applied to other fabrics for the localization of temperature and pressure stimuli.

In addition to being woven into 2D fabric webs, the optoelectronic fibers can also be constructed into other topologies for more complex functions. For example, a 3D closed-surface sphere constructed of photodetecting fibers woven into the sphere's longitudes and latitudes can be used to measure the illumination direction over 4π steradians [68]. A 2D fabric web can be used to extract the optical intensity distribution by virtue of a back-projection algorithm commonly used in computerized axial tomography [68]. The fidelity of the reconstructed image is improved by increasing the number of acquired projections. Furthermore, a more sophisticated task, namely the detection of both the amplitude and the phase of an optical wave front, has been realized using a dual-plane fabric array that is constructed using unprecedented optoelectronic fibers integrating eight cascaded photodetectors (Fig. 2d) [69], enabling non-interferometric lensless imaging (Fig. 2e)and f).

Fluorescence imaging has also been demonstrated using a single highly integrated fiber that combines an optical fiber core with two photodetectors situated at the tip of the hybrid fiber (Fig. 2g)and h) [60,70]. When the incident light guided by the optical fiber illuminates on a fluorescent material (Fig. 2i), the emission light can be immediately detected by the photodetectors made out of semiconducting nanowire-based optoelectronics (Fig. 2h). Scanning the fiber over the fluorescent object while simultaneously recording the photocurrent allows imaging without the use of optical lenses (Fig. 2j).

### Fabrics hear and speak

Many modern technologies such as communications, augmented reality, human–machine interfaces, robotics and healthcare rely on the detection and perception of audible sounds. Incorporating this capability into fabrics can endow them with sophisticated applicability and functions such as fabric-based sound localization, acoustic communications and health auscultation. The path towards achieving sensitive acoustic fabrics faces two major obstacles. First, traditional fabric materials in this regard are notorious in damping sound and second, piezoelectric fibers reported previously suffer from very low sensitivity in air necessitating a large areal fraction of active fibers thus interfering with the textile's desirable wearable properties [38].

**Figure 3.**
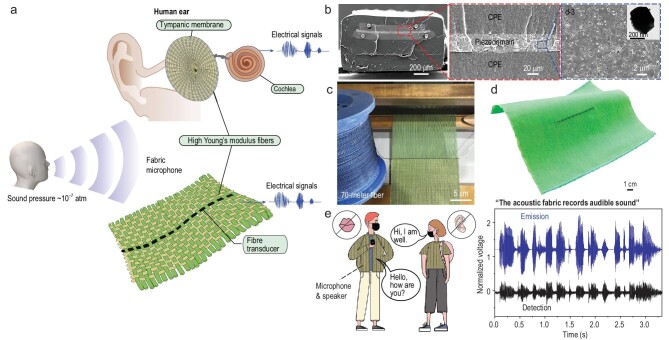
(a) Principles and design of the fabric microphone. The tympanic membrane is a four-layer construct of high-modulus collagen fibers, and it transduces sound pressure to mechanical vibrations of the middle-ear bones. Such vibrations are then transmitted to the cochlea of the inner ear wherein hair bundles are deflected to ultimately convert pressure waves into electrical signals (ionic) that are picked up by the nervous system. In analogy to the tympanic membrane, the fabric microphone is constructed of high-modulus twaron yarns woven into low-modulus cotton yarns. This fabric medium converts faint audible frequency sound pressure waves into low-order mechanical vibration modes. A single strand of woven piezoelectric fiber has a similar role to the cochlea in providing electrical output. (b) SEM micrographs of the piezoelectric fiber that consists of a piezoelectric nanocomposite sandwiched between two conducting polymeric electrodes, each of which is in contact with two copper wires. The whole structure is encapsulated in an elastomeric SEBS cladding. (c) Photograph of the piezoelectric fiber woven into a fabric. (d) Photograph of the fabric microphone. (e) Schematic of acoustic communication enabled by the fabric microphone and speaker. The speech ‘The acoustic fabric records audible sound’ emitted by one shirt is recorded by the other shirt. (a–e) Reproduced with permission [38]. Copyright 2022, Nature Publishing Group.

By drawing inspiration from the human auditory system, Yan *et al.* revealed principles, materials, design concepts and fabrication strategies that afford a scalable pathway to transform fabrics into sensitive audible microphones (Fig. 3a) [38]. The approach involves two important enabling steps. First is the construction of a fabric made of high-modulus textile fibers into the weft of a cotton (ubiquitous basis material for fabrics) warp. This design converts tenuous 10^−7^-atmosphere pressure waves at audible frequencies into lower-order mechanical vibration modes. Second, within that same fabric, a single specifically elaborately designed thermally drawn flexible piezoelectric fiber is woven, in compliance with the vibration of the fabric, and converts the mechanical vibrations into electrical signals [38].

The piezoelectric fiber consists of a piezocomposite layer composed of P(VDF-TrFE) loaded with BaTiO_3_ ceramic nanoparticles sandwiched between two carbon-based nanocomposite electrodes, each in contact with two copper wires. This assembly is encapsulated in an elastomeric styrene ethylene butylene styrene (SEBS) cladding (Fig. 3b). The combination of this soft cladding with the stiffer piezoelectric composite results in stress concentration in the active piezoelectric domain, efficiently converting mechanical energy into electrical energy. The P(VDF-TrFE) undergoes thermoplastic deformation while the BaTiO_3_ nanoparticles retain their morphologies unchanged during the draw, resulting in the formation of tiny voids around the BaTiO_3_ nanoparticles. This ferroelectret structure can provide one explanation for the high piezoelectric charge coefficient (d_31_) of ∼46 picocoulombs per newton [38].

The scalability of the thermal-drawing approach is well matched to the scale of industrial weaving processes (Fig. 3c). The piezoelectric fiber can be woven into acoustic fabrics using conventional weaving looms (Fig. 3d). Measuring the vibrational behavior of these fabrics can enable their transduction mechanisms in the audible frequency range. Thus, a Scanning Laser Vibrometer is used to measure the spatial displacement of the acoustic fabric while simultaneously extracting the electrical signals emanating from that same fabric in response to acoustic waves. These concurrent measurements establish the underlying physics of the fabric microphone: (i) the acoustic fabric with a high Young's modulus converts tenuous 10^−7^-atm pressure waves at audible frequencies into lower-order mechanical vibration modes; (ii) synergistic vibrational coupling occurs between the fabric medium and the acoustic fiber; (iii) longitudinal bending, rather than compression normal to the fiber surface, dominates the electrical output, revealing the critical contribution of the d_31_ piezoelectric charge coefficient; (iv) the longitudinal bending contour of the fiber is determined by conformal matching with the membrane/fabric modes. Modes that result in lower displacement and the fiber curving in different directions (high-order vibrational modes) lead to lower electrical output. The technology yields innovative acoustic fabrics capable of detecting and recording audible sounds with performance on a par with and even surpassing some conventional rigid, point microphones. Applications in sound-direction detection, acoustic communications and heart-sound auscultation demonstrate a wide spectrum of applicability of the fabric microphone.

Piezoelectric materials can not only convert mechanical energy into electrical energy, but also convert electrical energy into mechanical energy. Thus, in addition to sensing sound as a microphone (Fig. 3e), the fabric can also broadcast audible sounds as a speaker when a modulated AC voltage is input. A fabric that speaks could be very useful for people who are deaf or hearing impaired, playing music that improves people's sleep or noise cancellation [38].

In addition to sensing audible sounds, thermally drawn piezoelectric fibers immersed in water can also work in both the emission and reception modes in the ultrasound frequency range. The performance of the fiber can be improved via increasing the effective area of the piezo-domain. A fabric constructed of several fibers shows coherent interference and beam-steering capabilities [71]. Large-area, flexible and wearable ultrasound fabrics offer unusual possibilities in medical imaging, bone healing, focused ultrasound surgery and ocean exploration.

### Fabrics communicate

Speech communication enables the transfer of information among people. Acoustic fabrics with high sensitivities in the audible range introduced in the previous section are particularly useful for establishing communication between individuals who are either hearing-impaired or speech impaired, as illustrated in Fig. 3e. Despite suffering from a relatively low data rate and low signal-propagating rate, direct communication via audible sounds builds the foundation for the communication of our everyday lives.

**Figure 4.**
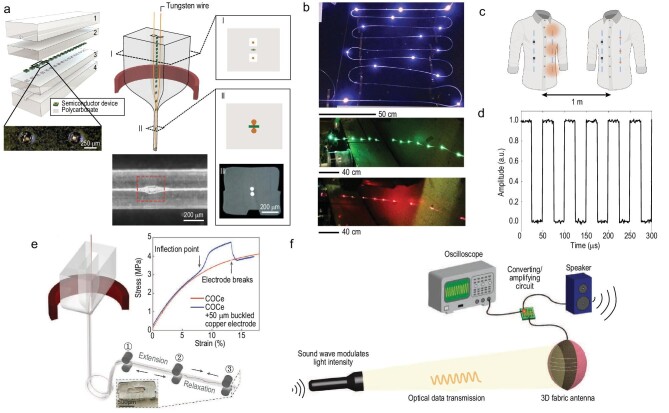
(a) Fabrication scheme for embedding high-performance microelectronic chips into the thermal-drawing platform. (b) Photographs of light-emitting fibers containing InGaN blue-color LEDs, InGaN green-color LEDs and AlGaAsP red-color LEDs. (c) Schematic of bidirectional communication system concept. Both shirts contain light-emitting and photodetecting fibers. The light-emitting fibers are modulated to transmit information that is being detected by the photodetecting fibers in the other garment, placed at a distance of 1 m from each other. (d) Experimental result of the photocurrent recorded by the photodetecting fibers incorporated into a fabric. The light was emitted from LED fibers embedded in another fabric located at a distance of 1 m from the photodetecting fabric. (a–d) Reproduced with permission [39]. Copyright 2018, Nature Publishing Group. (e) Strategy to harnesses the buckling instability in a metallic microwire for the fabrication of elastic fibers that are highly electrically conductive. (f) Optical fabric antenna for optical data communication. (e and f) Reproduced with permission [76]. Copyright 2022, Wiley-VCH.

The development of information technology heavily relies on high-speed and long-distance optical communication [72,73]. This requires the use of high-performance semiconductor diodes. The incorporation of them into fabric-grade fibers can enable fabric-based optical communication. However, the challenge associated with the mixing between typical semiconducting materials (e.g. silicon, germanium and indium gallium arsenide) and typical metallic electrodes during thermal drawing precludes the fabrication of semiconducting diodes of high quality in fibers [74,75]. A novel approach developed recently circumvents this challenge via packaging microchip-based semiconductor diodes into a polymer fiber while *in situ* encompassing electrical connectorization in the neck-down region during the draw (Fig. 4a) [39]. Light-emitting and photodetecting p–i–n diode fibers have thus been fabricated (Fig. 4b). Fabrics integrated with high-speed optical transmitters and receivers enable optical data communications (Fig. 4c)and d). This work demonstrates a new paradigm for realizing sophisticated functionalities in fibers and fabrics.

To further enhance the applicability of diode-based fabrics, it requires that the integrated fibers be soft and even stretchable. Two approaches enabling elastic fibers that are highly electrically conductive have been proposed [76]. The first approach harnesses the buckling instability in a metallic microwire that is included in a hollow channel of an elastomer fiber (Fig. 4e). The second approach relies on the simultaneous twisting of both the metallic microwire and elastomer cladding that yields a helical configuration. To create stretchable diode fibers, microscale diodes are first connected with metallic microwires, and then the entire chain is fed into the channel of the elastomer fiber during drawing. The metallic wires connecting the diodes are subsequently buckled using the two approaches described above, forming stretchable diodes fibers (Fig. 4e). Soft and stretchable fabric optical antennas are constructed by weaving these diode fibers into the

fabric. The fabric antenna is capable of receiving light signals modulated by sound waves and converting electrical signals into original sound, thus achieving long-distance optical-assisted acoustic communication (Fig. 4f) [76].

### Fabrics display

The ability to integrate light-emitting display functions into fabrics constitutes a compelling platform for fabric-based communications, human–machine interfaces, smart homes and innovative fashions. To enable their practical applications, fabric displays should possess excellent electroluminescent performance while retaining their traditional qualities, such as flexibility, machine-washability and conformability to the human skin. The incorporation of inorganic material-based light-emitting diodes into thermally drawn fiber also opens up an opportunity for realizing flexible and stretchable fabric displays as illustrated (Fig. 4b) [39]. These microchips are so small that they are imperceptible to wearers. The fabric preserves performance throughout 10 machine-wash cycles thanks to the robust electrical connectorization between the chips and conductive wires. However, the pixel spatial resolution of the fabric is limited due to the intrinsically large scale-down feature of the approach. A strategy to address this issue is to create each display pixel at the crossover points between the weft and warp yarns. The electric field formed between the electrodes in the yarns allows electroluminescent emission emanating from the zinc sulfide (ZnS) phosphor coated on the yarn surface at the crossover points. This enables a 6-meter-long, 25-centimeter-wide display fabric containing 5 × 10^5^ electroluminescent units spaced ∼800 micrometers apart [77].

### Fabrics diagnose health

Fabrics cover the most potentially valuable real estate in the world, i.e. the surface of the human body. This allows fabrics to be exposed to a variety of human vital signs including body temperature, pulse rate, respiration rate and blood pressure as well as physical activities and physiological state [78–81]. Smart fabrics working as large-area sensors allow users to monitor their healthcare conditions in a continuous, real-time and long-term manner, paving a novel way towards personalized healthcare.

**Figure 5.**
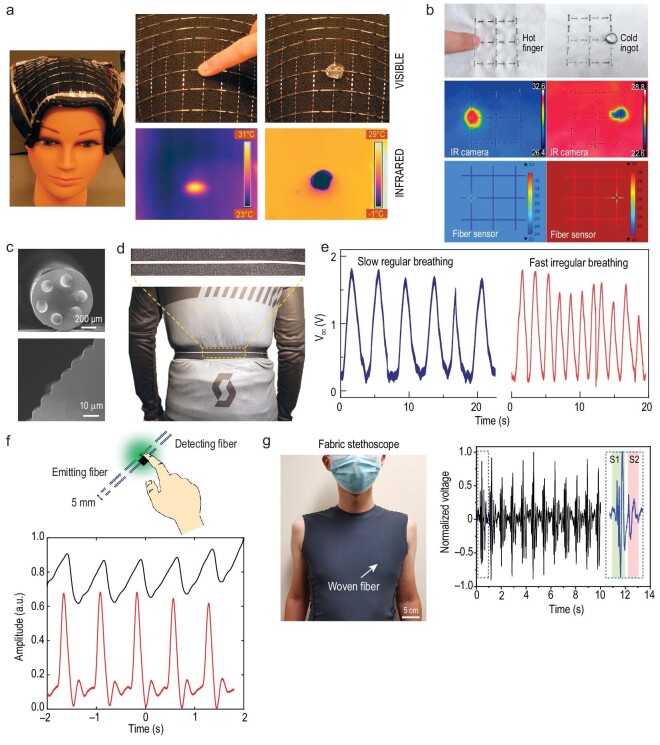
(a) Temperature mapping obtained using the thermal-sensing fabric constructed of an array of thermal-sensing fibers where the core elements are chalcogenide semiconducting glasses. Reproduced with permission [62]. Copyright 2006, Wiley-VCH. (b) Temperature mapping obtained using the thermal-sensing fabric constructed of thermoelectric fibers containing materials such as Bi_0.5_Sb_1.5_Te_3_ or n-type Bi_2_Se_3_. Reproduced with permission [63]. Copyright 2019, American Chemical Society. (c) SEM micrographs of the stretchable fiber integrating eight liquid-metal electrodes. The surface of the fiber is textured to enhance the triboelectric output. (d) The fiber is fixed on a stretchable belt, which is worn around the torso of a person. (e) The output voltage spectra recorded using the belt allow the quantitative assessment of breathing patterns. (c–e) Reproduced with permission [35]. Copyright 2020, Nature Publishing Group. (f) Schematic of a photoplethysmography pulse-measurement set-up using light-emitting (dashed line with green light) and photodetecting (dashed line with black square) fibers placed at a distance of 5 mm from each other. Experimental result of the current measured by the photodetecting fiber compared to the output of a gold-standard pulse sensor. Reproduced with permission [39]. Copyright 2018, Nature Publishing Group. (g) Fabric stethoscope monitors heart health. The shirt interfaces with the chest and can detect heart sounds, thus providing information about the cardiovascular system: it measures the heart rate and both the S1 and S2 components of the heart sound. Reproduced with permission [38]. Copyright 2022, Nature Publishing Group.

Body temperature reflects much information about health status. Endowing fabrics with the ability to sense temperature requires the integration of thermal-sensing fibers. One strategy relies on the use of chalcogenide semiconducting glasses whose resistances are temperature-dependent [62]. The resistance can be extracted by measuring the current–voltage curve of the fiber device. The weave structure of the fabric allows the localization of thermal excitation. The measurement precision reaches as high as 0.1°C (Fig. 5a). The second strategy is to incorporate thermoelectric materials such as Bi_0.5_Sb_1.5_Te_3_ or n-type Bi_2_Se_3_ into glass fibers with high softening temperatures (Fig. 5b) [63]. Thermoelectric materials can convert thermal energy into electrical energy by transforming infrared photons into current. When a temperature gradient is established in a fabric, the temperature of any position that is either higher or lower than that of the reference point can be detected by measuring the voltage potential between the hot and cold sides. Both the temperature distribution and the position of heat/cold source with a spatial resolution of millimeters can be achieved using such a fabric (Fig. 5b). The polycrystalline nature of the as-drawn thermoelectric core can be transformed into a single-crystal structure via a laser annealing approach, leading to drastically improved performance [82,83].

Many physiological processes such as heart beating, breathing, snoring and speaking generate mechanical vibrations or deformation of the skin. Piezoelectric and triboelectric generators represent the fundamental technology for the detection of these signals via converting mechanical energy into electrical energy. A triboelectric fiber constructed of a Geniomer cladding, an elastomer with a higher negative triboelectric polarity compared with poly tetrafluoroethylene (PTFE), encapsulating six liquid-metal alloy (Galinstan) electrodes (Fig. 5c) is woven into a stretchable belt worn around the torso (Fig. 5d), which efficiently discriminates irregular breathing from regular breathing of the wearer (Fig. 5e) [35]. The diode fibers discussed previously enable photoplethysmography pulse measurement. The measured pulse rate agrees well with the result obtained using gold-standard sensors (Fig. 5f) [39]. The acoustic fabric enabled by the piezoelectric fiber discussed previously is integrated into a vest, which allows users to monitor the resting heart rate, the louder S1 sound and the weaker S2 sound, potentially together with the characteristics of the splitting of S1 as well as the splitting of S2, previously unachievable with thin-film devices (Fig. 5g) [38]. Users are able to hear their heart sounds very clearly with such a vest via playing back the recorded electrical signals. The ability to monitor physiological signals using everyday fabrics represents the most promising technology towards personalized healthcare.

### Fabrics detect motion

**Figure 6.**
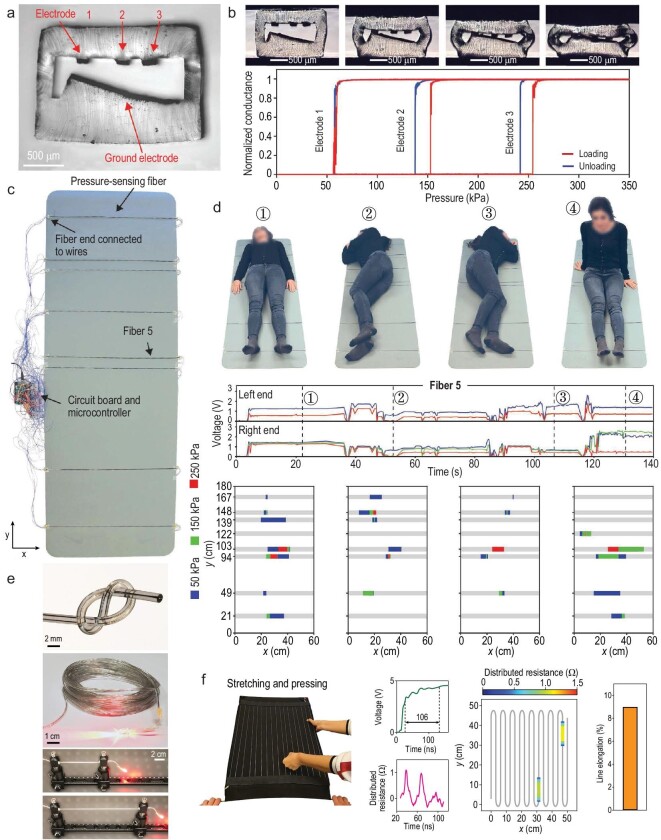
(a) Optical micrograph of the fiber cross section. Three small conductive cPE electrodes are arranged opposite to a large inclined electrode in an SEBS cladding. (b) Optical micrographs of the fiber cross section under increasing pressure. The consecutive contact of the electrodes results in an abrupt increase in the corresponding conductance at distinct pressure levels. (c) Eight pressure-sensing fibers are integrated on a gymnastic mat for human body posture and motion monitoring. (d) The mat measures the positions of a volunteer lying on the mat. Signals collected from all fibers were translated into positions using the calibration curves. (a–d) Reproduced with permission [40]. Copyright 2020, Wiley-VCH. (e) Photograph of the soft transmission line with two liquid-metal conductors. Both of them remain electrically conductive but insulated from one another over extended lengths. The conductivity remains unchanged even when the line is being stretched to 100% elongation. (f) A fabric integrating one transmission line is harnessed for multiplexed deformation sensing when it is simultaneously exposed to the two modes of deformation: stretching and pressing. (e and f) Reproduced with permission [26]. Copyright 2020, Nature Publishing Group.

Smart fabrics with motion-sensing capabilities offer exciting opportunities for healthcare monitoring, human–machine interactions and sports training [27]. The detection of mechanical stimulation such as compression, elongation, bending and torsion based on capacitive, piezoresistive, piezoelectric and triboelectric effects has been demonstrated. A micro-electromechanical fiber that consists of two electrically conductive nanocomposite layers separated by an air gap can detect and localize touch or compression with high accuracy along the entire length of the fiber [84]. Compared with typical metallic electrodes (e.g. Cu or Ag), the nanocomposite electrodes are relatively resistive. Applying a voltage at the same extremity of the two electrodes causes a voltage drop that is associated with the distance between the contact point (pressure point) and fiber extremity. Thus, measuring the voltage drop allows the extraction of the position of a local touch. The integration of such fibers into fabrics would enable fabric pianos that play music via simple finger touching. Furthermore, the fiber can be soft and even stretchable. Incorporating liquid metals such as Gallium or Gallium-based alloys into the fiber realizes elongation sensing [55]. The gauge factor of such a strain fiber sensor is comparable to the best liquid-metal-based devices. When two electrodes are integrated into the fiber, forces as low as 0.01 N can be measured with the capacitive effect. The robustness of these fibers is demonstrated by stable electrical properties over 10^5^ stretching cycles. When a 3D assembly of a liquid-metal electrode and four nanocomposite electrodes are integrated within the fiber, various functions such as the detection of compression and the magnitude, position and even direction of the stimulus can be simultaneously achieved using such a single fiber [55]. This impressive technological breakthrough brings tremendous opportunities in various fields. For example, the integration of multiple fibers with a similar structure into a gymnastic mat enables monitoring of body position, posture and motion (Fig. 6a–d) [40]. Moreover, the soft fiber containing deformable liquid-metal electrodes can work as transmission lines based on electrical time-domain reflectometry (Fig. 6e) [26]. Integrating these fibers into fabrics allows the detection of the mode, magnitude and position of multiple simultaneous pressing and stretching events (Fig. 6f). This drastically contrasts with typical methods that exploit networks of sensors specific to one type of deformation.

### Fabrics harvest energy

**Figure 7.**
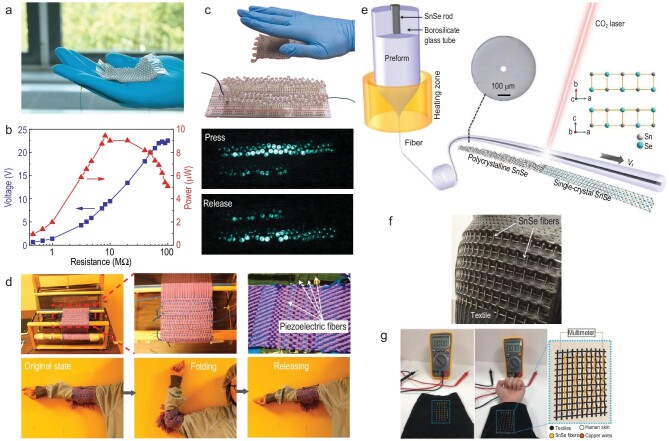
(a) Photograph of the triboelectric fabric constructed of a continuous and long triboelectric fiber that consists of a liquid-metal core encapsulated in a SEBS cladding. (b) Output signals versus the resistance of external loads. (c) Demonstration of 100 LEDs lit up by the fabric under tapping. (a–c) Reproduced with permission [35]. Copyright 2020, Nature Publishing Group. (d) The piezoelectric fabric harvests energy from the elbow motion. Reproduced with permission [61]. Copyright 2017, American Chemical Society. (e) Schematic of thermal-drawing SnSe fiber and post-draw laser recrystallization process. The insets show the cross-sectional microscope image of SnSe fiber and the crystal structure of SnSe. (f and g) A large-area fabric constructed of SnSe fibers converts human body heat into electricity. (e–g) Reproduced with permission [53]. Copyright 2020, Wiley-VCH.

Energy and power sources are fundamentally important for the deployment of smart fabrics. To augment the applicability and sustainability of smart fabrics, their self-powering and self-storage capabilities are greatly required. Smart fabrics harvesting energy via triboelectric, piezoelectric, thermoelectric fiber technology have already been established [35,53,61,85]. A 36-cm^2^ triboelectric fabric integrating super-elastic liquid-metal-based fibers triggered by simple hand tapping can generate an open-circuit voltage and short-circuit transferred charges as high as 490 V and 175 nC (Fig. 7a–c), respectively, which is on a par with the performance of the state-of-the-art 2D planar triboelectric nanogenerators with similar dimensions [35]. Because of the super-elastic nature of both the elastomer and the liquid metal, the fabric exhibits extraordinary long-term robustness and durability under severe compression and impact. A piezoelectric fabric integrating PVDF-BaTiO_3_ fibers worn on a human elbow can generate open-circuit voltages of up to ∼10 V and short-circuit currents of 5−15 nA under a 90° bend−release action of the elbow (Fig. 7d) [61]. The performance can be further improved by engineering the structure of the piezoelectric domain of the fiber and weaving more fibers into the fabric. A thermoelectric shirt that contains several SnSe fibers and worn on the body can generate an output voltage of as high as 30 mV because of the temperature difference between the skin and the environment (Fig. 7f)and g) [53]. The SnSe in the fiber exhibits a single-crystal feature with rock-salt structure that is thermally constructed using a CO_2_ laser crystallization scheme (Fig. 7e). The thermoelectric figure of merit ZT of the single-crystal fiber reaches 2 at 862 K. Single-crystal SnSe has been reported as a record holder of high-performance thermoelectric materials. Conventional methods for the fabrication of single-crystal SnSe lead to bulky, rigid and planar form factors. The work demonstrated here constitutes a new paradigm for the development of flexible, wearable SnSe-based thermoelectric electronics.

### Fabrics store energy

The ability to directly store the energy harvested from the surroundings via the aforementioned mechanistic deployment into the fabric would allow the powering of entire fabric electronics using the fabric itself instead of external heavy and rigid devices. High-performance fiber batteries or supercapacitors that are flexible, impermeable and machine-washable are thus needed. Conventional methods used to fabricate fiber-based energy-storage devices require multiple steps, such as coating, twisting and bonding, and small-scale multifilament assembly, hindering the performance of the resulting fiber and the fabrication scalability. Achieving thermally drawn fiber energy-storage systems faces several major challenges. First, the electrolyte and electrode materials should have similar viscosities such that they can undergo a laminar flow where the cross-section geometry is preserved during the preform-to-fiber transition. It requires the co-flowing of all components, the physical separation of electrodes as well as intimate contact between electrodes and the electrolyte. Second, typical active materials for batteries or supercapacitors either have very high melting points or break down prior to thermal drawing. Third, the high temperature during thermal drawing might activate chemical reactions between fiber materials, causing the destruction of the device structure.

**Figure 8.**
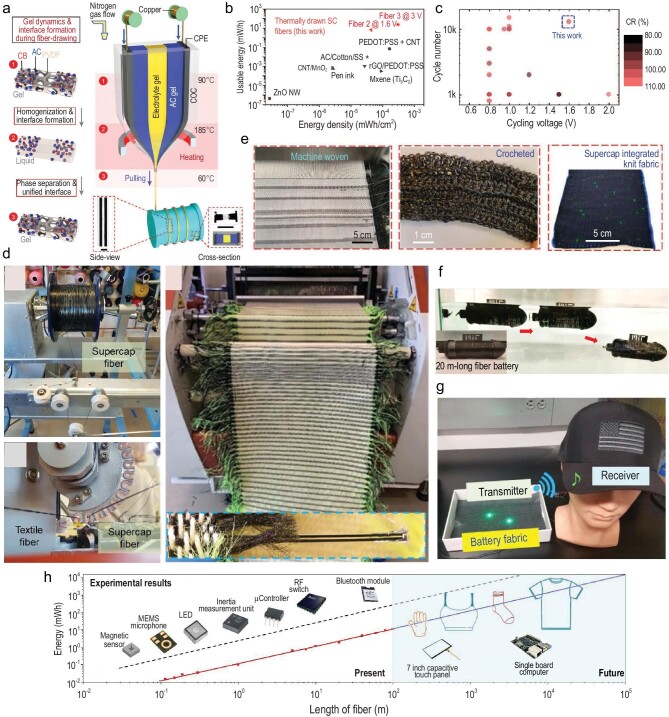
(a) Principles and fabrication of thermally drawn supercapacitor fibers. A designed thermally induced phase separation is introduced into the fiber-drawing process for producing interconnected porous structures of both electrodes and electrolyte. (b) Comparison of usable energy and areal energy density between the thermally drawn supercapacitor fibers and the state-of-the-art fiber cells reported in the literature. (c) Cycle number, cycling voltage and capacitance retention comparison of fiber-shaped supercapacitors. (d) Large-scale, continuous manufacturing of supercapacitor fabrics by machine weaving of the thermally drawn supercapacitor fibers. (e) Supercapacitor fabric products. (a–e) Reproduced with permission [36]. Copyright 2020, Wiley-VCH. (f) The operation of submarine is powered by 20-m fiber battery casing. (g) A 100-m fiber battery-powered LiFi fabric sample as a next-generation communication fabric. (f and g) Reproduced with permission [65]. Copyright 2022, Elsevier. (h) Development trend of the fiber energy-storage system. The left side (white background) shows energy values of thermally drawn energy-storage fibers (experimental results) with respect to the length. Current fibers can operate various electronic devices including magnetic sensors, microphones, motion sensors, switches, Bluetooth modules, etc. In the future (blue background), these fibers will be embedded into the garments such as gloves, underwear, socks and outer clothing that can operate many advanced electronic devices such as displays and computers. Reproduced with permission [36]. Copyright 2020, Wiley-VCH.

These challenges in the fiber-drawing platform have been addressed recently via a series of materials and processing breakthroughs, which have enabled the first ultra-long thermally drawn supercapacitor fiber and battery fiber [36,65]. To ensure laminar flow without the mixing of different materials, the gel matrix of both the electrolyte and the electrodes is prepared by using a common composition. To maximize the surface area that facilitates high storage density per unit fiber length as well as high ionic transport properties, an elegantly designed thermally induced phase separation is introduced into the fiber-drawing process for producing interconnected porous structures of both electrodes and electrolyte (Fig. 8a). To achieve stable performance over the entire fiber length, which has been regarded as an outstanding challenge in energy-storage fibers, highly conductive metallic microwires are incorporated into large-area polymeric nanocomposite electrodes, which ensures fast axial electron transport.

A single draw produces a hundreds-of-meter-long fiber supercapacitor or battery that is fully functional [36]. The supercapacitor fiber of a

five-component cell architecture exhibits a maximum areal energy density of 306 μWh cm^−2^ and usable energy of ≈24 mWh. Its capacitance remains stable over 13 000 cycles of charge–discharge at a current density of 2 mA cm^−2^ and cycling voltage of 1.6 V as well as 100 machine-washing cycles (Fig. 8b)and c). The fiber can be woven into large-area fabrics using automated weaving machines in a continuous way (Fig. 8d)and e). The resulting fabric can simultaneously power microcontrollers and LEDs integrated into the same fabric for free-space optical data communications. A wide range of other electronic components such as sensors, microphones and switches can also be powered using such a fabric [36]. The fiber battery of 140 meters in length displays a discharge capacity of ∼123 mAh and discharge energy of ∼217 mWh [65]. The performance of the fiber battery increases proportionally with the fiber length without limitation relevant to length. A 20-meter-long fiber can power a submarine drone underwater (Fig. 8f). The fiber is woven into a large-area fabric of 135 × 120 cm^2^ via an automated weaving machine, which enables the powering of a suite of fabric electronics such as microphones, pre-amplifiers, transistors and diodes integrated into the same fabric (Fig. 8h). Such a fabric can receive audio signals optically and transform the signal back to the electrical domain. The received electrical signal can then be fed to a receiver that can output to a speaker, thereby realizing a fabric communication system entirely powered by the fiber battery (Fig. 8g) [65]. We envision that, in the near future, a single fiber with enhanced performance will power a new generation of smart fiber, fabric and 3D-object electronic systems (Fig. 8h).

### Fabrics store and process data toward computation

Digital computers are progressing from bulky, heavy versions to mobile, lightweight, flexible form factors for increased interface with humans. Such human-interfaced digital systems, also known as wearable computers, are being increasingly used for applications in physiological monitoring and medical intervention, human–machine interactions, as well as robotics and human assistance. However, constrained by the necessity to convince prospective users to carry additional devices, the most well-developed technologies are broadly available only in a small number of highly specific planar form factors, limiting widespread usage. Moreover, producing such systems, by combining and connecting large numbers of sensors, memory units and other digital components, with flexible properties and large-scale manufacturing abilities remains a challenge. A desirable technology to address this goal is functional fabrics that are worn continuously and have an a-priori advantage of being in intimate contact with large surface areas of the human body, presenting a significant opportunity to harvest, store and even analyse relevant untapped information from the human body.

**Figure 9.**
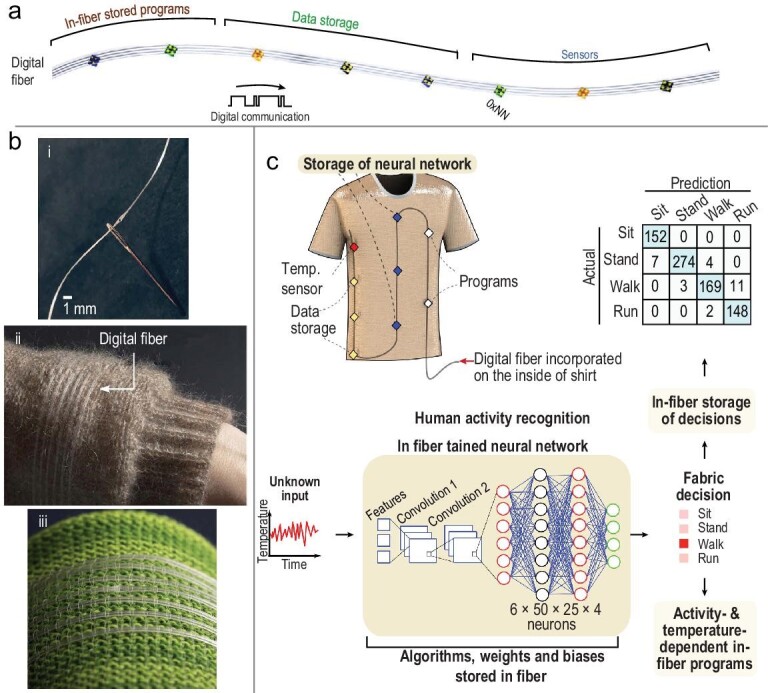
(a) Illustration of the thermally drawn digital fiber integrating chips of different functionalities such as memory, communication and sensing. (b) Integration of the digital fiber (i) through a needle, (ii) in the sleeve of a sweater and (iii) in a cotton-based fabric. (c) Schematic of the shirt with a woven digital fiber that integrates sensors, data storage, programs and a neural network stored within its memory devices. This digital fabric is capable of running a neural network that recognizes what the wearer is doing in real time without any human intervention. The accuracy of human activity recognition by the in-fabric convolutional neural network (CNN) is shown by the prediction table, indicating an average accuracy of 96.4%. (a–c) Reproduced with permission [33]. Copyright 2021, Nature Publishing Group.

Thermal drawing has been harnessed to process inorganic silicon-based digital microelectronics to create a continuous flexible polymeric fiber strand, spanning tens of meters with digital capabilities including analog-to-digital sensing, digital communication, memory and in-fiber data analytics (Fig. 9a) [33]. Precise control over the positions and angles of discrete particulates within the fluid polymer flow and usage of a soft–hard polymer combination allow continuous electrical connection between meters-long microwires and the entire array of microscale discrete digital devices. The entire ensemble of distinct devices is accessible and independently operated through a single connection at the edge of the fiber, overcoming the perennial single-fiber single-device limitation. A fabric incorporating such a digital fiber (Fig. 9b) is used to collect and store the data of surface body temperature over multiple days along with the physical activities associated with the temperature patterns. A neural network with 1650 neuronal connections is trained on the basis of the temperature–time–activity correlation and is subsequently stored in the same fiber. When presented with an unknown temperature–time data set, the fiber infers the type of physical activity with an accuracy of 96% based on a single temperature-measurement point (Fig. 9c). The ability to measure and store digital physiological data in a fiber that also contains inference algorithms presents intriguing opportunities for large-scale fabrics that sense, memorize, learn and feed back towards computation.

## CONCLUSION AND OUTLOOK

With the rapid development of the field of thermally drawn fibers, increasingly functional materials, structures and devices are incorporated into the fibers [56,86–91], forming a ‘Moore's law’ analog in fibers and fabrics. The broad applicability of these intelligent fibers and fabrics in the sensing of various stimuli (e.g. light, heat, sounds, chemicals, compression, elongation, bending, torsion, etc.), motion and healthcare monitoring, actuation, communication, display, energy harvesting and storage, data storage and analysis have all been demonstrated, presenting intriguing opportunities for lots of emerging technologies. Despite these advancements, many challenges relevant to scientific and technological questions still remain.

Materials build the foundation for the performance of fiber and fabric devices and their real-world applications. Although various functional materials have been harnessed in the thermal-drawing platform, the processing requirements imposed on this technique preclude the use of many materials. Recent progress on the incorporation of prefabricated miniaturized semiconductor devices such as photodetectors, photodiodes and memory chips into thermally drawn fibers provides a unique avenue towards realizing high-performance fabrics. Semiconductor nanowires [92] and 2D semiconductors [93–95] might endow fibers/fabrics with novel performance and functions because these materials can be fully integrated post-draw via simple coating, deposition or printing techniques. Besides the direct exploitation of high-performance materials and devices, enhancing the performance of fiber materials via control over materials’ structure on the micro- and nanoscale levels remains a compelling route [96–98], calling for greater emphasis on the fundamental research of materials for fibers and fabrics. A great example of this exploration is the creation of a porous piezoelectric structure via leveraging the difference in the thermoplastic deformation properties of a piezoelectric polymer matrix and embedded ceramic nanoparticles, enabling high-performance acoustic fibers and fabrics with extraordinary sensitivities [38].

The thermal-drawing technique is an extraordinary scalable fabrication method—>100 million kilometers of optical fiber are produced every year. However, the preparation of preforms is separated from the drawing of fibers. Making the entire process continuous is crucial for the mass production of smart fabrics. Thermally drawn fibers exhibit exquisite micro- and nanostructures, which dictates the complexity of preform preparation. More advanced techniques should be developed to construct preforms with sophisticated architectures in a more continuous and cost-effective manner. One strategy is to exploit 3D printing technology that enables direct digital manufacturing of fiber preforms with complex cross-sectional architectures with great simplicity, autonomy and efficiency [99]. The resulting fibers can be incorporated back into the 3D printing feedstock in a recursive manufacturing way to further enhance the complexity and functionality of the fiber. Thanks to rapid progress in the field, there are many manufacturers for fiber-draw towers that can be customized depending on the specific requirements of users, making this technique feasible and universal in both industry and academia.

The functionality of smart fabrics is becoming increasingly versatile. The era of computing fabric is around the corner driven by tremendous research momenta. Digital functions including digital sensing, data storage, digital communication, data analysis and digital programming are essential for fabric computation. The incorporation of microelectronic chips into the thermal-drawing platform presents an appealing breakthrough towards this research goal, which is in contrast with the current prevalent scheme—wireless data management where data are wirelessly transmitted to a nearby computing station (e.g. laptop and mobile phone) that performs remote data analysis. Moreover, artificial intelligence (AI) and machine-learning (ML) technologies are also required to integrate with fabric computers to deliver more advanced intelligence for the wearers. For example, AI- and ML-enabled smart fabrics can directly analyse a large number of bio-signals collected from the human body and identify hidden markers for diseases and illnesses, which might be helpful for early warning of cardiovascular abnormalities, COVID diseases, etc. Finally, an autonomous closed-loop fabric system that simultaneously enables healthcare monitoring, data analysis, visualization and communication, on-demand therapeutic abilities, powering technologies as well as alert modules without external human intervention is an appealing direction.

To ensure robust practical applications of fibers and fabrics, novel materials with high mechanical reliability and compliance should be exploited to enhance their tolerance to sophisticated deformations and washability. For example, encapsulating the supercapacitor fiber with an exquisitely engineered elastomeric layer endows the device with superior elasticity and extreme bendability [65]; therefore, the supercapacitor fabric woven from these fibers exhibits nearly identical electrochemical performance after 100 cycles of machine-washing. On the other hand, a novel structural design can be applied on the fiber and fabric surface to achieve self-cleaning ability. Thus, a fabric might spontaneously clean itself of organic stains with light when a nanostructured photoactive layer is constructed on its surface, thereby bypassing the regular machine-washing [100].

To conclude, the ‘Moore's law’ for thermally drawn fibers represents a unique platform for emerging intelligent fabric technologies. Future advance in fiber materials, architecture, fabrication, system integration as well as in-fiber AI and ML will push the limits of current fabrics, enabling the transformation of fabrics from goods into sophisticated computing platforms for value-added services. We hope that the progress and challenges discussed in this review will constitute a source of inspiration and motivation for both researchers already immersed in the field and newcomers to further overcome roadblocks and encompass new developments in the field. An era of fabric computation is emerging!
